# Individual Oligogenic Background in p.D91A-*SOD1* Amyotrophic Lateral Sclerosis Patients

**DOI:** 10.3390/genes12121843

**Published:** 2021-11-23

**Authors:** Giulia Gentile, Benedetta Perrone, Giovanna Morello, Isabella Laura Simone, Sebastiano Andò, Sebastiano Cavallaro, Francesca Luisa Conforti

**Affiliations:** 1 Institute for Biomedical Research and Innovation, Department of Biomedical Sciences, National Research Council (CNR), 95126 Catania, Italy; giulia.gentile@cnr.it (G.G.); giovanna.morello@irib.cnr.it (G.M.); sebastiano.cavallaro@cnr.it (S.C.); 2 Medical Genetics Laboratory, Department of Pharmacy and Health and Nutritional Sciences, University of Calabria, 87036 Rende, Italy; benedetta.perrone90@gmail.com (B.P.); sebastiano.ando@unical.it (S.A.); 3 Neurology Unit, Department of Basic Medical Sciences, Neurosciences and Sense Organs, University of Bari “Aldo Moro”, 70121 Bari, Italy; isabellalura.simone@uniba.it; 4 Centro Sanitario, Department of Pharmacy and Health and Nutritional Sciences, University of Calabria, 87036 Rende, Italy

**Keywords:** p.D91A-*SOD1*, zygosity, NGS targeted-gene panel, individual oligogenic background

## Abstract

The p.D91A is one of the most common ALS-causing *SOD1* mutations and is known to be either recessive or dominant. The homozygous phenotype is characterized by prolonged survival and slow progression of disease, whereas the affected heterozygous phenotypes can vary. To date, no genetic protective factors located close to *SOD1* have been associated with the mild progressive homozygous phenotype. Using Next Generation Sequencing (NGS), we characterized a small cohort of sporadic and familial p.D91A-*SOD1* heterozygous (*n* = 2) or homozygous (*n* = 5) ALS patients, to reveal any additional contributing variant in 39 ALS-related genes. We detected unique sets of non-synonymous variants, four of which were of uncertain significance and several in untranslated regions of ALS-related genes. Our results supported an individual oligogenic background underlying both sporadic and familial p.D91A cases irrespective of their p.D91A mutant alleles. We suggest that a comprehensive genomic view of p.D91A-*SOD1* ALS patients may be useful in identifying emerging variants and improving disease diagnosis as well as guiding precision medicine.

## 1. Introduction

Amyotrophic lateral sclerosis (ALS) represents the third most common neurodegenerative disease, characterized by the progressive adult-onset degeneration of upper and lower motor neurons [1]. There are two main forms of ALS, familial (FALS) and apparently sporadic (SALS) accounting for about 10% and 90% of cases, respectively [2]. This complex disease is caused by the interplay of causative genetic factors (monogenic or oligogenic) and risk factors (genetic and non-genetic) [3]. 

The first ALS-related gene described was superoxide dismutase 1 (*SOD1*), whose mutations affect about 12% of FALS and 1% of SALS [1]. The most common mutation affecting *SOD1* and causing ALS is the substitution of alanine for aspartate at position 91 of exon 4, called p.D91A (also known as p.D90A; dbSNP^155^ ID rs80265967; NM_000454.5, c.272A>C) [4]. Prevalence of p.D91A in ALS cases varies globally and is distinctly absent in some populations (https://gnomad.broadinstitute.org/, accessed on 22 October 2021). In particular, this is the most prevalent variant in Europe [5]. 

Despite the extensive evidence demonstrating the pathogenicity of p.D91A in *SOD1* and according to the variant interpretation and assertion criteria of ACMG guidelines [6], this variant is still reported in ClinVar with conflicting interpretations of pathogenicity. However, the p.D91A variant is reported as a risk allele, resulting in disease when biallelic or in combination with another risk factor, by the more recent ClinGen curation database [7].

Although all the mutations affecting *SOD1* are dominant, p.D91A can also be recessive [2] and the disease status may arise from heterozygous or homozygous mutant alleles, respectively. Indeed, in Scandinavia the p.D91A allele is a variant associated with recessive inheritance [8,9], while in many other countries dominant inheritance was also associated to the disease [10,11,12]. In particular, in Nordic countries p.D91A-*SOD1* is reported with a polymorphic frequency of 2.5% [13], rendering this polymorphism a risk factor in those countries.

All p.D91A-homozygous ALS patients show a phenotype characterized by a slower course of disease and not always associated with respiratory failure or cognitive issues [1,9,14,15], while the clinically affected p.D91A-heterozygotes present variable clinical signs and disease progression. There are no affected p.D91A-heterozygotes among homozygous pedigrees [4] and there is limited literature showing p.D91A-*SOD1* affected heterozygous patients in multiple members of an ALS family [16,17].

Moreover, p.D91A carriers belong not only to FALS but also to SALS cases, and *SOD1* haplotypes show a common ancestor with a shared Scandinavian haplotype of rare alleles in both homozygous and heterozygous patterns [13,18]. Thus, two phenotype-explaining hypotheses have been proposed, the presence of a protective genetic modifier on the Scandinavian haplotype or a co-segregating contributing variant together with the p.D91A haplotype outside Scandinavia [19]. 

An updated literature search for previously reported p.D91A *SOD1*-related phenotypes [20] revealed the presence of at least three groups of patients with differences in disease progression rate and survival time, without fully identifying potential genetic modifiers or contributing variants in addition to the p.D91A zygosity (Table 1). 

The first group of patients displays a slowly evolving phenotype linked to the p.D91A-homozygous genotype [1,9,14,15,16] with no other identified genetic modifier responsible for the mild phenotype. In addition, a p.D91A-homozygous patient has been recently described having vocal cord impairment, which is not a typical clinical sign associated with this genotype [15]. 

The second group of patients, concerning the heterozygous pattern of zygosity, includes cases with compound heterozygous mutations (p.D91A/p.D96N; p.D91A/p.D90V), showing a lower limb site of onset and a slow progressive phenotype with a variable disease duration, ranging from 7 to 28 years [21,22,24]. In addition, five clinically affected p.D91A-heterozygous cases with slow progression of disease were reported: one patient with a negative family history for the disease [23], another belonging to a large family carrying the TDP-43 p.G298S mutation [17], and three individuals of the same family [16]. However, it is not clear if other *SOD1* variants were investigated in these cases. 

The third p.D91A *SOD1*-related phenotype, shown in Table 1, is characterized by an aggressive evolution of the disease and is found in individuals described carrying a dominant p.D91A variant co-segregating with ALS [4]. In this group falls the case of a p.D91A-heterozygous affected carrier showing TDP-43 aggregates, with a family history of ALS and other neurodegenerative diseases [25]. Few other case reports described coexistence of TDP-43 inclusions with dominantly inherited *SOD1* variants since these aggregates are neuropathological hallmarks of ALS-FTD and SALS patients [25,26,27,28].

Furthermore, the p.D91A-heterozygous mutation plus the pathogenic *C9ORF72* repeat expansion or the variant of uncertain significance (VUS) *UBQLN2*-Q460R [29] were already described in patients associated with the ALS-FTD phenotype [19,30].

Differences in the genotype–phenotype correlations delineated above may have considerable therapeutic implications. Indeed, recruitment for antisense therapy was recently discouraged in p.D91A-heterozygous affected carriers after finding evidence of one case showing TDP-43 aggregates as autoptic findings [25]. 

Although no further variants have been identified in the conserved region surrounding *SOD1* that may explain the mild progressive phenotype in homozygous mutation carriers, the existence of contributing genetic factors in other DNA regions cannot be ruled out [31].

Based on these premises, in this study we investigated by targeted NGS the presence of additional variants in 39 ALS-related genes in SALS and FALS patients carrying the p.D91A-*SOD1* heterozygous or homozygous mutation, with the aim to reveal any other contributing variant able to explain the homozygous mild phenotype.

## 2. Materials and Methods

### 2.1. Patients

Informed consent was obtained from all the participants included in the study. ALS patients (three familial and four sporadic cases, four women and three men) from southern Italy unrelated families, diagnosed with ALS according to the El Escorial criteria [32], were previously recruited at our institution and genetically defined as carriers of the p.D91A-*SOD1* heterozygous or homozygous mutation by Sanger sequencing analysis, as previously reported [14,33]. Clinical features and known genetic background of patients described in this study are shown in Table 2.

In our cohort, patients affected by the biallelic p.D91A variant, mainly showed a prolonged survival unlike p.D91A heterozygous affected carriers. ALS patients without *SOD1* mutations, together with individuals affected either by a motor neuronal (MN) phenotype or other neurodegenerative diseases not associated to ALS and belonging to our Southern Italian reference population were used as control samples (C1-C8) since we were not interested in pleiotropic effect [34]. Our filtering strategy aimed to identify rare and polymorphic variants able to synergistically act with the already known causative mutation, so data were normalized for two different types of confounding factors, such as genetic background and overlapping phenotypes at the same time.

The study was approved by the local Ethics Committee of Azienda Ospedaliero Universitaria of Bari N 1025.

### 2.2. NGS Analysis

Genomic DNA quality and quantity were evaluated using standard agarose electrophoresis and Qubit™ Fluorometer (Invitrogen, Waltham, MA, USA). Based on quantitative results, samples were normalized to 50 ng/μL to be used as an input in targeted NGS sequencing analysis. Deep sequencing analysis was performed using a custom ALS-related gene panel including genes known to be associated or possibly associated with ALS and overlapped phenotypes. In detail, we targeted the coding regions of 39 ALS-related genes with at least 25 bp of intronic flanking regions, together with the promoter region of the following subset of genes: *SOD1*, *TARDBP*, *FUS*, *ANG*, *ALS2*, *TBK1*, *SPG11*, *PFN1*, *TUBA4A*, *SETX*, *VCP*, *MATR3*, *VAPB*, *CCNF*, *NEK1*, *HNRPA1*, and *ERBB4* (Supplementary Table S1) [35]. Libraries were prepared using the custom Ion AmpliSeq kit (Life Technologies, Carlsbad, CA, USA), and sequencing analysis was run on an Ion Torrent Personal Genome Machine™ (PGM™) sequencer (Thermo Fisher Scientific, Carlsbad, CA, USA).

To set the bioinformatic pipeline, we followed the best practice consensus recommendations developed by the College of American Pathologists and the American Medical Informatics Association [36]. Primary and secondary data analysis were performed using the Torrent Suite (Thermo Fisher Scientific, Carlsbad, USA), with the Human genome [19] to align sequences and a Germline low stringency variant caller setting. Tertiary level data analysis was carried out using Partek Flow software build version 10.0.21.0302 (Partek Inc., St. Louis, MO, USA. Variant annotation was performed using Ensemble transcript release 75, SnpEff, VEP.84 databases. Variant frequencies in ALS patients and controls belonging to the project MinE database, were annotated using the project MinE data browser (http://databrowser.projectmine.com/, accessed on 20 April 2021), whose current dataset contains WGS data from 4366 ALS cases and 1832 controls [37]. Variants were then pre-filtered for SNP low stringency quality parameters (FAO > 2, FDP > 6, QUAL> 20, STB < 0.9) to filter out false positives and retain >99% of true positives calls (a strategy optimized for amplicon-based semiconductor sequencing) [38]. Variants with a QD < 1 were also filtered out based on our experience on SNV false positive calls and the observation that true positive calls have high mean coverage and quality by depth values [39]. Variant prioritization was carry out filtering for Read Depth ≥ 20X (minimum read depth for germline variants calling), and for MAF—European Ancestry Population—Freq < 0.5. We further removed intronic variants and synonymous variants not affecting canonical splice sites. The same NGS and bioinformatic pipelines were applied either on patients (P1-P7) or controls (C1-C8) to compare and filter patients’ variants. Comparison between patient and control variants were performed using the Summarize cohort mutations by merging pairs option in Partek Flow. Variant classification was performed querying VarSome (https://varsome.com/, accessed on 20 April 2021), a search engine for human genomic variation freely available and implemented to automatically classify and report the variant classification according to ACMG guidelines [40], and ClinVar (https://www.ncbi.nlm.nih.gov/clinvar/, accessed on 20 April 2021) [41]. We also used CADD GRCh37-v1.6 (https://cadd.gs.washington.edu/snv, accessed on 24 October 2021) [42] prediction ranking for deleteriousness of variants, without setting an arbitrary cut-off for our disease model, since p.D91A variant is reported having a CADD score of 9.481, which is below the scaled scores of 10 (predicted to correspond to the 10% most deleterious substitutions in the human genome). Inferential statistics was not conducted because of the small sample size. Descriptive statistics for variant frequencies was calculated in our case series, in our South Italian reference population [33], as well as inferred by the related population databases.

### 2.3. eQTLs

Expression Quantitative Trait Loci (eQTL) analysis, helpful to understand the effect of genetic variations on the transcriptome in healthy post-mortem tissues donors, was performed using Genotype-Tissue Expression GTEx Portal v.8 (www.gtexportal.org, accessed on 24 October 2021) [43]. The eQTL for each of the 19 variants of interest was calculated for five tissues of interest (whole blood, brain cortex, brain frontal cortex, spinal cord, and skeletal muscle) using GTEx eQTL Calculator, generating a *p*-value for each variant-gene pair T-statistics in an eQTL. T-test results were corrected using Bonferroni correction test.

## 3. Results

Data obtained from deep sequencing analysis of p.D91A-*SOD1* patients are reported in detail in supplementary data. In particular, coverage analysis and alignment quality parameters are shown in Supplementary Tables S1 and S2, respectively. Results obtained after pre-filtering for variant quality and prioritization for germline read depth and frequency in the European population showed only already known variants (Supplementary Table S3). We obtained a p.D91A average read depth across the seven ALS patients of 616X, and a mean quality by depth of 8.4 and 38.3 for heterozygous and homozygous, respectively.

**Figure 1:**
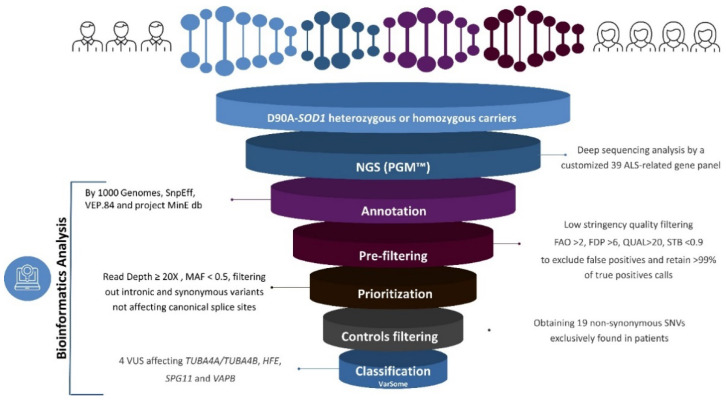
Workflow of the variant prioritization strategy adopted. The figure was created editing funnels and healthcare infographics provided by https://infograpia.com/, accessed on 12 July 2021.

The variant prioritization strategy adopted (Figure 1) showed the presence of 19 SNVs in p.D91A-*SOD1* patients (Table 3). Using the *VarSome* tool, four variants were classified as a VUS (rs45488900, rs41266793, rs139334167, rs76708676). 

In Table 4 we list the co-occurrence of variants in ALS-associated genes in each patient: 19 variants were exclusively found in patients and not in controls; two variants, in *PON1* and/or *GRN*, instead, were detected in both patients and controls (except for P1 and C1). Only two variants were shared by two or three patients, including one segregating with the genotype zygosity. This was the case of *TUBA4A/TUBA4B* rs45488900, shared by two p.D91A-homozygous SALS patients of our cohort, P4 (Het) and P5 (Hom). The entire list of annotated variants detected in patients and controls is available in Supplementary Table S4. 

To investigate the possible relation between variants detected by our analysis and gene loci affecting gene expression, particularly for untranslated region variants, we also calculated their potential effect on gene expression through their mapping on eQTLs. Data retrieved by GTEx Portal v.8 and corrected by a Bonferroni correction test (Supplementary Table S5) showed a tissue-specific effect for three out of 19 variants queried in non-diseased tissues of interest (rs11555696, 183 rs34099167, and rs118018900).

## 4. Discussion

In this study, we performed targeted NGS analysis in a small group of south Italian ALS patients, previously genetically characterized as p.D91A carriers, hypothesizing that genetic factors in other ALS-related genes, in combination with the p.D91A-*SOD1* variant, may contribute to the different disease phenotypes in homozygous and heterozygous cases. 

Recently, Sanger sequencing analysis performed in 997 ALS patients from southern Italy by our research group, revealed that 2% of patients had SOD1 mutations [33]. In particular, the frequency of p.D91A affected individuals represented 0.8% of all ALS cases diagnosed (0.6% p.D91A-hom and 0.2% p.D91A-het). These data are in line with the frequency of this mutation reported by other Italian research groups [57]. 

Previous reports demonstrated the absence of a neuroprotective factor in the genomic region near *SOD1* in p.D91A-homozygous ALS patients, suggesting the existence of a putative protective factor modulating the phenotype located elsewhere in the genome [31].

The present investigation showed that p.D91A-heterozygous and -homozygous ALS cases do not contain a genetic modifier near *SOD1*, nor near ALS-linked genes, highlighting the presence of unique variant gene sets in each patient (Table 4). A similar conclusion was recently published in a study investigating the A90V-*SOD1* mutation in SALS patients, suggesting that additional genetic variants could contribute to disease penetrance [24].

We identified 19 non-synonymous variants, including four of uncertain significance, in ALS-D91A carriers. Most substitutions (13/19) exclusively found in p.D91A patients were in non-coding regions of ALS-related genes, while the remaining (6/19) were missense mutations without any clear evidence of pathogenic effects. Although large genes (i.e., *SETX* or *NEK1*) have more chance to accumulate rare variants, the 19 variants identified in genes related to ALS were exclusively found in patients and not in controls. However, no clear genotype–phenotype correlation was established due to the small sample size.

All but one patient (P1) showed the presence of variants already identified as risks factors for neurodegenerative diseases [56,57,58], rs662 (*PON1*) and rs5848 (*GRN*) (Table 4). Previous studies inconsistently suggested an effect of *PON1* SNPs on ALS susceptibility, and rs662 was associated with bulbar onset and reduced survival in ALS cases very recently [55]. However, all the patients carrying this risk factor in our cohort showed a spinal onset of the disease. The *GRN* rs5848 polymorphism was reported in Alzheimer’s Disease (AD) and Parkinson’s Disease (PD) patients as risk factor for ubiquitin- and TDP-43 -positive frontotemporal degeneration [59]. Interestingly, this genetic variant lies in the binding-site for the miR-659 of the 3’UTR of *GRN* and may alter gene regulation [59].

Two variants classified as VUS were already described, the rs139334167 in *SPG11* and the rs45488900 in *TUBA4A*. The missense variation affecting *SPG11* was previously reported in a case of PD [44], while the rs45488900 affects *TUBA4A* but also the upstream region (n.-1456G>T) of *TUBA4B*. The encoded protein of the latter, was found differentially over-expressed in post-mortem pre-frontal cortex samples of patients affected by atypical ubiquitin-positive frontotemporal lobar degeneration, characterized by ubiquitin and FUS positive inclusions, while *TUBA4A* was down-expressed in the cerebellum of the same group of patients when compared to controls [58]. Interestingly, rs45488900 was shared by p.D91A-homozygous SALS patients showing a slow course but with a different clinical picture of the disease (Table 2; Table 4). Due to the small number of homozygous SALS in our cohort, no inference on the possible role of this variant in association with the phenotype could be made, but this aspect remains noteworthy and deserves to be explored in a larger number of cases. We did not find any literature reports describing two other variants classified as VUS. These substitutions, positioned in the 5’UTR of *HFE* (rs41266793) and in the 3’ UTR of *VAPB* (rs76708676), were detected in the homozygous case P3, and in the P5 patient with the longest observed disease duration (22 years) (Table 3).

Further investigating the potential influence of identified variants on gene expression by eQTL analysis, we also observed that two polymorphisms, rs34099167 and rs11555696, were associated with deregulated expression levels of *DCTN1* and *NEK1* in some of the tissues considered (Supplementary Table S5). Interestingly, the altered expression of these two genes was already found by our research team using unsupervised clustering of gene expression in motor cortex samples, identifying two transcriptome-based SALS subgroups of patients [60]. In particular, *NEK1* was found down-regulated in one cluster of patients while the second one was characterized by increased expression of *DCTN1* and reduced levels of *SOD1* [60]. NEK1 belongs to NIMA-related serine/threonine kinases family and is involved in mitochondrial membrane regulation, DNA damage response, ciliogenesis and maintenance of the cytoskeleton network [29], while DCTN1 is a motor protein involved in dynein-mediated axonal retrograde transport and ciliogenesis [61]. Moreover, dynactin (Dctn1) acting with overexpressed dynamitin (Dctn2), was shown to produce a late-onset progressive motor neuron disease inhibiting axonal transport in transgenic mice [62]. In our data, rs34099167 and/or rs11555696 were detected in 3 out of 7 patients, both homozygotes and heterozygotes, showing different clinical features, although no correlation between phenotype and genotype was established (Table 4). 

Recently, the analysis of two different cohorts, with a majority of apparently sporadic cases, showed an oligogenic basis of ALS associated with earlier age of disease [63,64]. Differences in genotype-phenotype correlations would have considerable therapeutic implications. ALS is a devastating pathology in which multiple variants cooperate in influencing disease onset, severity or duration and, until now, no truly effective treatment exists [65]. Thanks to recent efforts to selectively treat *SOD1*-related ALS patients, ASO therapies designed to knock-down the expression of the gene have emerged [66]. To this regard are of particular interest two ongoing phase 3 studies using intrathecally administered ASO Tofersen, an orphan drug capable of reducing SOD1 protein levels [67]. To ensure the appropriate recruitment of patients to clinical trials it appears evident the importance to establish if p.D91A mutation is pathogenic in the heterozygous state and if other contributing factors influence the phenotype.

## 5. Conclusions

Our study suggests the possibility that additional genetic factors contribute to the individual oligogenic basis of p.D91A-*SOD1* carriers. In particular, all patients, except for P2 carrying only risk factors, showed an oligogenic pattern in line with the model proposed for ALS etiopathogenesis in which mutations in two or more genes are required to develop the disease, but they are not all necessarily truly pathogenic [1,19]. Increasing the number of sequenced p.D91A patients could be useful in identifying emerging genetic factors and improving disease diagnosis, as well as guiding precision medicine.

## Figures and Tables

**Table 1:** p.D91A *SOD1*-related phenotypes.

p.D91A *SOD1* zygosity	Survival Time	Progression Rate	Phenotype	Contributing Risk Factors Proposed	References
Hom	More than two years	Slow	Spinal ALS *	Contributing variants mitigating the phenotype not yet identified	[1,9,14,15,16]
Het	More than two years	Slow	Spinal ALS	Heterozygous compound in *SOD1*	[16,17,21,22,23,24]
Het	About two years	Fast	Variable forms of ALS **	Contributing variants or TDP-43 inclusions not yet identified	[4,5,25]

Hom = homozygous; Het = heterozygous; * with or without respiratory failure and/or cognitive issues; ** including bulbar onset.

**Table 2:** Clinical and genetic data of p.D91A-*SOD1* patients.

FALS or SALS	Sample ID	Mutant Allele	Gender M/F	Site of Onset	Age of Onset (yrs)	Disease Duration (yrs)
FALS	P1	Hom	F	LL	68	n/a
FALS	P2	Hom	M	LL	49	8 ^a^
FALS	P3	Hom	F	LL	46	2.2 ^a^
SALS	P4	Hom	F	LL	55	8.4 ^b^
SALS	P5	Hom	M	UL	33	22 ^b^
SALS	P6	Het	M	LL	52	2.5 ^c^
ALS	P7	Het	F	LL	54	2 ^a^

^a^ Patient died; ^b^ alive in April 2021; ^c^ no more follow-up since 2013. All the data have been inferred from patient medical records and from previously published data [14,33]. FALS = familial amyotrophic lateral sclerosis; SALS = sporadic amyotrophic lateral sclerosis; Hom = homozygous allele variant; Het = heterozygous allele variant; SNV = single nucleotide variation; Gender = male/female; S = UL for upper limbs and LL for lower limbs; n/a = data not available. The allele frequency percentages for both variants are: 0.001432 (gnomAD v2 1.1.exomes), 0.00207 (gnomAD v2 1.1.genomes), and 0.0004 (Genome Project databases).

**Table 3:** Variants identified in p.D91A-*SOD1* patients by targeted NGS are annotated with frequency and classification.

Gene	V	rs ID	VA	VF	MAF	VarSome	ClinVar	Proj. MinE	CADD PHRED Score	Ref.
*DCTN1*	c. * 21C>T	rs11555696	3′ UTR	0.14	0.022720/ 0.02188	LB	B	n/a	8.217	[44]
*TUBA4A*	c.227-74C>T	rs45488900	Intron	0.28	-/0.12853	VUS	n/a	n/a	5.309	[45]
*TUBA4B*	n.-1456G>T		Upstream		n/a	n/a	n/a	n/a	5.309	n/a
*NEK1*	c.2255A>G; p.Glu752Gly	rs34099167	Missense	0.4	0.14032/ 0.09304	B	B	n/a	25.1	n/a
*NEK1*	c.1388C>T; p.Ala463Val	rs34540355	Missense	0.14	0.035127/ 0.03250	B	B/LB	0.0593/0.0603	16.28	n/a
*HFE*	c.-48C>G	rs41266793	5′ UTR	0.14	-/-	VUS	n/a	n/a	0.233	n/a
*FIG4*	c. * 29G>A	rs10659	3′ UTR	0.14	0.046277/ 0.07829	B	B	n/a	0.408	n/a
*SETX*	c. * 849G>T	rs74975459	3′ UTR	0.14	-/0.01150	B	B	n/a	3.561	n/a
*SETX*	c.59G>A; p.Arg20His	rs79740039	Missense	0.14	0.009062/ 0.00653	B	B	0.00882/0.00764	0.166	[46,47,48]
*SPG11*	c.7069C>T; p.Leu2357 Phe	rs139334167	Missense	0.14	-/0.00083	VUS	CIoP	0.00183/0.00164	25.8	[49]
*SPG11*	c.2083G>A; p.Ala695Thr	rs78183930	Missense	0.14	0.012527/ 0.01897	B	B	0.0121/0.0150	26.8	[49,50,51,52,53]
*PG11*	c.1108G>A; p.Glu370Lys	rs77697105	Missense	0.14	0.016740/ 0.02196	B	B/LB	0.0182/0.0194	21.6	n/a
*FUS*	c. * 910C>T	rs118018900	Downstream	0.14	0.04772/ 0.02543	B	B	n/a	1.015	n/a
*PFN1*	c.-342T>C	rs148770753	5′ UTR	0.14	-/0.00848	B	n/a	n/a	10.19	n/a
*VAPB*	c. * 753C>G	rs6070466	3′ UTR	0.14	0.01155/ 0.00452	B	B	n/a	15.82	n/a
*VAPB*	c. * 1265G>C	rs7400	3′ UTR	0.14	0.05550/ 0.09272	B	B	n/a	14.85	n/a
*VAPB*	c. * 2819A>G	rs74568509	3′ UTR	0.14	0.04862/ 0.07642	B	B	n/a	0.673	n/a
*VAPB*	c. * 4520T>C	rs763514	3′ UTR	0.14	0.05098/ 0.10229	B	B	n/a	0.83	n/a
*VAPB*	c. * 6182C>T	rs76708676	3′ UTR	0.14	0.02834/ 0.01132	VUS	B	n/a	0.515	n/a
*APEX1*	c. * 2A>T	rs17112002	3′ UTR	0.14	0.003300/ 0.00373	LB	n/a	n/a	5.682	[54]

* V, variant; VA, variant annotation; VF, variant frequency out of 7 p.D91A patients; MAF, minor allele frequency in Exome Aggregation Consortium (ExAC) and Genomes Aggregation Database (GnomAD); VarSome, the human genomic variant search engine—(B) benign, (LB) likely benign, (VUS) uncertain significance; Project MinE variant browser, a database providing information on genetic variations found in WGS of ALS patients and controls—AF ALS cases/AF controls; ClinVar, archive of interpretations of clinically relevant variants—(B) benign, (LB) likely benign, (CioP) conflicting interpretations of pathogenicity; n/a, data not available. CADD PHRED score, combined annotation dependent depletion.

**Table 4:** Co-occurrence of variants in ALS-associated genes in each patient.

Patient ID	FALS or SALS	p.D91A SOD1	Genes	HGVSc/HGVSp	Zygosity	rs ID	Ref.
P1	FALS	Hom	DCTN1	c. * 21C>T	Het	rs11555696	
			NEK1	c.2255A>G; p.Glu752Gly	Hom	rs34099167	
P2	FALS	Hom	PON1	c.575A>G; p.Gln192Arg	Het	rs662	[55]
			GRN	c. * 78C>T	Het	rs5848	[56]
P3	FALS	Hom	HFE	c.-48C>G	Het	rs41266793	
			FIG4	c. * 29G>A	Het	rs10659	
			GRN	c. * 78C>T	Het	rs5848	[56]
P4	SALS	Hom	TUBA4A/TUBA4B	c.227-74C>T/ n.-1456G>T	Het	rs45488900	
			SPG11	c.7069C>T; p.Leu2357 Phe	Het	rs139334167	
			VAPB	c. * 753C>G	Het	rs6070466	
			PON1	c.575A>G; p.Gln192Arg	Het	rs662	[55]
			GRN	c. * 78C>T	Hom	rs5848	[56]
P5	SALS	Hom	TUBA4A/TUBA4B	c.227-74C>T/ n.-1456G>T	Hom	rs45488900	
			NEK1	c.2255A>G; p.Glu752Gly	Het	rs34099167	
			PFN1	c.-342T>C	Het	rs148770753	
			VAPB	c. * 6182C>T	Het	rs76708676	
			APEX1	c. * 2A>T	Het	rs17112002	[54]
			GRN	c. * 78C>T	Het	rs5848	[56]
P6	SALS	Het	NEK1	c.1388C>T; p.Ala463Val	Het	rs34540355	
			SPG11	c.2083G>A; p.Ala695Thr	Het	rs78183930	[49,52]
			SPG11	c.1108G>A; p.Glu370Lys	Het	rs77697105	
			PON1	c.575A>G; p.Gln192Arg	Het	rs662	[55]
			GRN	c. * 78C>T	Het	rs5848	[56]
P7	SALS	Het	NEK1	c.2255A>G; p.Glu752Gly	Het	rs34099167	
			SETX	c. * 849G>T	Het	rs74975459	
			SETX	c.59G>A; p.Arg20His	Het	rs79740039	[47]
			FUS	c. * 910C>T	Het	rs118018900	
			VAPB	c. * 1265G>C	Het	rs7400	
			VAPB	c. * 2819A>G	Het	rs74568509	
			VAPB	c. * 4520T>C	Het	rs763514	
			PON1	c.575A>G; p.Gln192Arg	Het	rs662	[55]

* V, variant.

## Data Availability

The data presented in this study are available in the results and supplementary material sections.

## Supplementary Materials

The following are available online at https://www.mdpi.com/article/10.3390/genes12121843/s1, Table S1: Quality metrics and coverage analysis parameters; Table S2: The table illustrates the alignment quality parameters (to hg19) related to the samples analysed in the same run; Table S3: Variants per patient; Table S4: The entire list of annotated variants detected in both patients and controls in availabale; Table S5: Variants associated with eQTL effects in different tissues.
